# Biosynthesis of cerium and gadolinium nanoparticles by *Pseudomonas putida* KT2440 enables sustainable rare earth elements utilization

**DOI:** 10.1038/s41598-026-59096-4

**Published:** 2026-06-23

**Authors:** Paz García-García, Elena Alonso-Fernandes, Cristina Serrano-Pelejero, Andrés González-Vega, Laura Castro, Adrián Rodríguez, Eduardo Díaz, Manuel Carmona

**Affiliations:** 1 Biotechnology Department, Centro de Investigaciones Biológicas Margarita Salas-CSIC, Madrid, Spain; 2 Department of Material Science and Metallurgical Engineering, Facultad de Químicas, Universidad Complutense de Madrid, Madrid, Spain; 3 Biotechnology Departament, Centro de Investigaciones Biológicas-CSIC, Madrid, Spain

**Keywords:** Rare earth elements, Lanthanides, Metallic nanoparticle, Biomining, Biotechnology, Circular economy, Biochemistry, Biological techniques, Biotechnology, Chemistry, Microbiology, Nanoscience and technology

## Abstract

Lanthanides (Ln), a group of 15 rare earth elements (REEs), are critical for advanced technologies, although their conventional extraction and processing are environmentally unsustainable. Here, we present a microbial platform based on *Pseudomonas putida* KT2440 for the eco-friendly recovery and transformation of Ln, introducing a key methodological innovation: the use of a resting cell system to circumvent the pervasive issue of abiotic lanthanide–phosphate precipitation. This approach enables controlled investigation of Ln biomineralization under mild conditions. Mechanistically, the results showed that Ln recovery proceeds via rapid cell-surface adsorption, followed by surface-templated nucleation and extracellular mineralization. This process leads to the formation of well-defined biogenic nanoparticles primary identified as CePO₄ and GdPO₄. Structural analyses reveal nanorod morphologies, while functional characterization shows that CePO₄ nanoparticles retain photoluminescent properties and GdPO₄ nanoparticles preserve paramagnetic behavior. Compared to conventional chemical synthesis, this biosynthetic strategy eliminates toxic reagents and energy-intensive steps, yielding biocompatible materials with controlled size and morphology. Our findings establish *P. putida* KT2440 as an efficient and sustainable platform for Ln recovery and functional nanoparticle production, providing a foundation for scalable green alternatives to traditional Ln processing.

## Introduction

The lanthanides (Ln), comprising the fifteen chemically similar elements from lanthanum (La) to lutetium (Lu), are collectively categorized, along with yttrium (Y) and scandium (Sc), as rare earth elements (REEs). Despite the term “rare”, REEs are relatively abundant in the Earth’s crust. However, their diffuse geological distribution and low ore concentrations make extraction technically and economically challenging^1^. Ln are strategic materials, essential for the manufacturing of high-performance magnets, light-emitting phosphors, rechargeable batteries, catalysts, and contrast agents for medical imaging. Moreover, they are integral to green technologies such as wind turbines, electric vehicles, and hydrogen fuel cells, positioning them at the forefront of the transition toward sustainable energy^2,3^. The surging global demand for REEs has prompted serious environmental and geopolitical concerns. Conventional Ln mining and refining rely on energy-intensive techniques such as acid leaching, solvent extraction, and thermal processing, which generate substantial toxic waste and contribute to environmental degradation. Additionally, these methods often result in significant greenhouse gas emissions and present risks to water and soil quality. The environmental footprint, coupled with vulnerabilities in international supply chains and concerns over resource depletion, has intensified the search for alternative, sustainable strategies for REE recovery and utilization^4^. On the other hand, Ln-based nanoparticles (LnNPs) have emerged as powerful platforms in biomedicine and catalysis due to their unique optical, electronic, and magnetic properties. For example, cerium oxide nanoparticles exhibit remarkable redox activity with applications in oxidative stress modulation, while gadolinium nanoparticles are highly valued as contrast agents in advanced imaging modalities due their paramagnetic properties. However, the conventional chemical synthesis of such nanomaterials is often associated with significant environmental concerns, including the release of toxic solvents, high energy consumption, and the generation of hazardous by-products. These drawbacks underscore the urgent need for greener, sustainable synthetic routes that preserve functionality while minimizing ecological impact. In this context, microbial biotechnology offers a promising and eco-friendly alternative for LnNPs production^5^.

Certain microorganisms isolated from natural environments possess the ability to bind, transport, or transform Ln, leading to a deeper understanding of the biochemical roles of Ln in biology^6,7^. For instance, several methylotrophic bacteria have been shown to utilize Ln as essential cofactors in methanol and other short-chain alcohol oxidation reactions, facilitated by Ln-dependent alcohol dehydrogenases^8^. These biological capabilities open novel pathways for applications in bioremediation, bioleaching, and the sustainable recovery of REEs from low-grade ores or industrial waste streams. Although Ln are involved in important biological processes as trace elements^9^, at elevated concentrations they can exert toxic effects on living organisms. Ln ions may inhibit calcium uptake in bacteria^10^, compete with calcium for binding to cellular proteins, and block calcium-permeable cation channels^11^. Their bioavailability and toxicity largely depend on their chemical form: soluble salts such as sulfates, bromides, nitrates, chlorides, and perchlorates are readily absorbed by biological systems^12^, while insoluble forms like oxides, sulfides, carbonates, hydroxides, oxalates, or phosphates exhibit limited bioaccessibility^13^ and display reduced toxicity^14^. In any case, the toxicity profile of Ln is complex and varies across the series, typically decreasing with increasing atomic number, though exceptions have been observed for the heaviest Ln (thulium, ytterbium, and lutetium), which have shown increased toxicity^7,15^.

The production of LnNPs by bacteria is a field largely unexplored. It has been described the sequestration of Ln ions and the formation of insoluble complexes by growing bacteria, most probably involved in the mitigation of Ln toxicity^16,17^. This study explores the potential of *Pseudomonas putida* KT2440 as a biological platform for LnNPs biosynthesis. *P. putida* KT2440 is a non-pathogenic, Gram-negative soil bacterium known for its extraordinary metabolic versatility and environmental resilience. It has been widely employed in diverse biotechnological applications, including the degradation of xenobiotics, resistance to heavy metals, tolerance to oxidative stress, and the bioproduction of functional nanomaterials^18,19^. The complete genome sequence of KT2440 is available^20^, and genome-scale metabolic models and sophisticated genetic engineering tools have been developed^21^. Importantly, *P. putida* KT2440 lacks virulence determinants and is considered a biosafety-compliant strain suitable for industrial and environmental use^22^. Although *P. putida* KT2440 is not a methylotrophic bacterium, it can metabolize volatile organic compounds containing alcohol and aldehyde functional groups, thanks to its periplasmic pyrroloquinoline quinone (PQQ)-dependent alcohol dehydrogenases, PedE and PedH^23^. PedE is calcium-dependent, whereas PedH is specifically activated by Ln^23,24^. The presence of Ln not only serves as a cofactor for PedH but also influences the transcriptional regulation of both dehydrogenases, effectively modulating substrate oxidation pathways^25^. A major focus of this work is the investigation of *P. putida* KT2440 intrinsic ability to biosynthesize LnNPs, a promising feature but limited on its mechanistic understanding and optimization. Traditional chemical synthesis of metallic NPs is associated with high energy demands, the use of hazardous organic solvents or extreme reaction conditions^26^, and the fact that residual chemical reagents may adhere to the surface of nanoparticles, potentially reducing their biocompatibility and applicability in medical or environmental contexts^27^. In contrast, microbial synthesis of nanoparticles represents a greener and safer alternative. Bacteria can serve as biological nanofactories, often yielding nanoparticles with high purity, uniform morphology, and low cytotoxicity, features that make them suitable for biomedical and environmental applications^28,29^.

In this study, we evaluate the capabilities of *P. putida* KT2440 to produce CeNPs and GdNPs, as a proof of concept of LnNPs production. The production of LnNPs in growing cultures is extremely challenging since the high phosphate content in standard growth media facilitates Ln precipitation and sequesters bioavailable phosphate^30^. To overcome these limitations, we performed the LnNPs production in resting cell conditions, i.e., using cells that remain metabolically active without growing. Unlike actively proliferating cultures, resting cells redirect cellular resources away from cell division and biomass formation and towards the bioconversion of substrates, thereby increasing the overall yield and specificity of target products^31,32^. In addition, resting cells can be used under controlled conditions where energy and cofactor regeneration are maintained, allowing for prolonged reaction times without the necessity of continuous nutrient supplementation. Finally, resting cell-based processes are particularly advantageous for the production of compounds that are toxic to actively growing cultures, as the absence of cell division reduces sensitivity to inhibitory effects. The LnNPs bioproduced were then purified and their physico-chemical properties analyzed. Our findings offer a framework never explored so far for the development of environmentally friendly methods for LnNPs production.

## Results and Discussion

### Ce and Gd toxicity on *P. putida* KT2440 resting cells

**Fig. 1:**
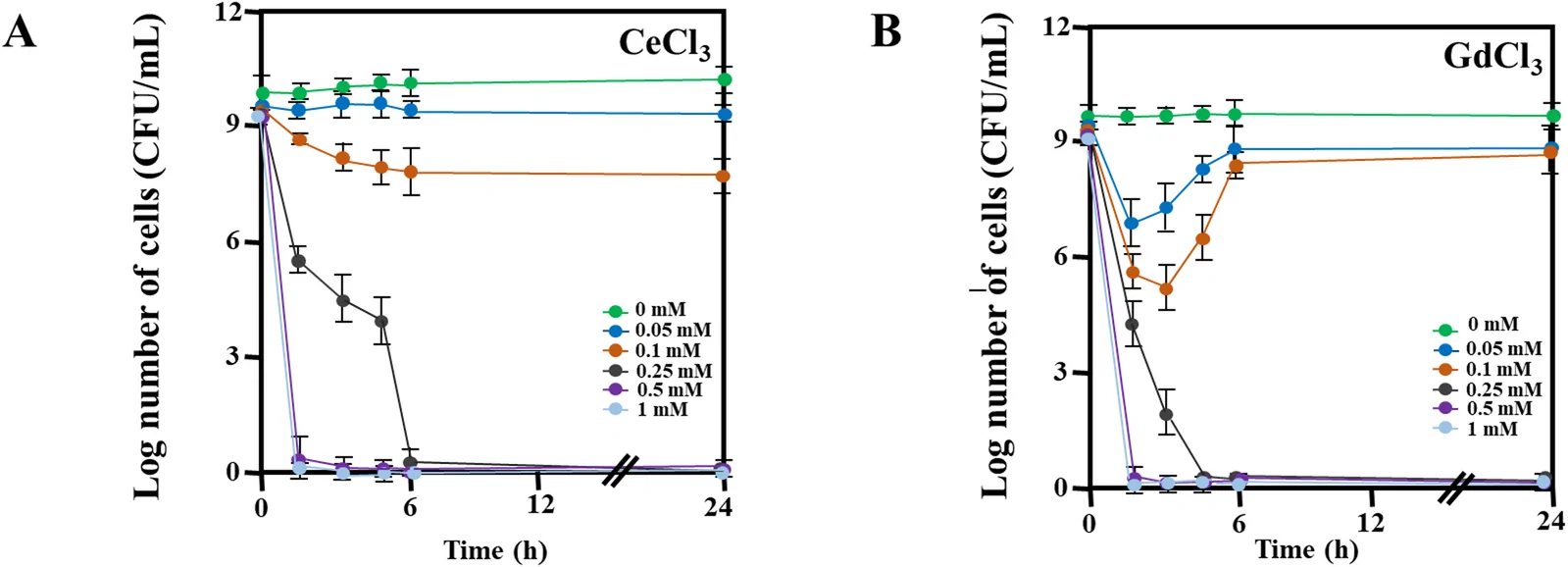
**Ce and Gd toxicity on** ***P. putida*** **KT2440.** Time course of the viability of *P. putida* KT2440 in saline solution supplemented with CeCl_3_ (**A**) or GdCl_3_ (**B**) at concentrations of 0 mM (green), 0.05 mM (blue), 0.1 mM (orange), 0.25 mM (grey), 0.5 mM (purple) and 1 mM (light blue). Error bars indicate the standard deviations of three independent experiments.

To establish the maximum Ce (as representative of a light Ln) and Gd (as representative of a heavy Ln) concentration tolerable by *P. putida* KT2440 we first assessed bacterial viability to Ce and Gd exposure under conditions mimicking NPs biosynthesis. As mentioned in the Introduction, Ln form insoluble complexes with phosphate, which is abundant in standard bacterial growth media, therefore a saline buffer system (0.85% NaCl) was used for NPs production minimizing precipitation side effects during resting cells. Cells were initially cultivated in LB medium at 30 °C, transitioned to minimal medium, and subsequently harvested, washed, and resuspended in saline solution as detailed in the Materials and Methods section. Ce^3+^ and Gd^3+^ were added at concentrations ranging from 0 to 1 mM, and viable cell counts were determined at 0, 3, 4, 5, 6, and 24 h post-treatment. Notably, viability decreased as a function of increasing atomic number, with significant toxic effects observed after 3 h of exposure, while light Ce caused only moderate reductions in cell viability at 0.1 mM, Gd promote growth disturbance above 0.1 mM and near-complete loss of viability at concentrations of 0.25 mM (Fig. 1).

The antibacterial properties of Ln were first noted in the mid-20th century, with reports describing their biphasic effects: stimulatory at micromolar levels and inhibitory at higher concentrations^33,34^. The cytotoxicity of Ln has been attributed to their ability to mimic calcium, disrupting calcium-dependent signaling, inducing phosphate depletion, and compromising membrane integrity^35^. Additionally, several Ln catalyze the formation of reactive oxygen species (ROS), further exacerbating oxidative stress in microbial cells^36,37^. A conventional understanding posits a decreasing toxicity trend of Ln with increasing atomic number^7^. Recently, Gorniak et al. reported that while light Ln (La, Ce, Nd) had limited inhibitory effects on *P. putida* KT2440, heavier lanthanides such as Gd significantly impaired growth^25^. It is crucial to mention that previous toxicity assessments were largely conducted under actively growing conditions. Under such scenarios, Ln typically present as trivalent cations (Ln³⁺), can precipitate as insoluble hydroxides at neutral pH unless complexed^30,38^. Furthermore, the high phosphate content in standard growth media facilitates Ln precipitation and sequesters bioavailable phosphate, confounding toxicity measurements. To overcome these limitations, we evaluated toxicity under phosphate-free, resting cell conditions. Our findings are congruent with those reported under growth conditions^25^, reinforcing the notion that Ln toxicity patterns persist across physiological states. To our knowledge, this is the first systematic investigation of Ce and Gd toxicity using *P. putida* KT2440 in resting state, a relevant condition for biotechnological applications such as LnNPs biosynthesis.

### Kinetics of Ce transformation by *P. putida* KT2440

**Fig. 2:**
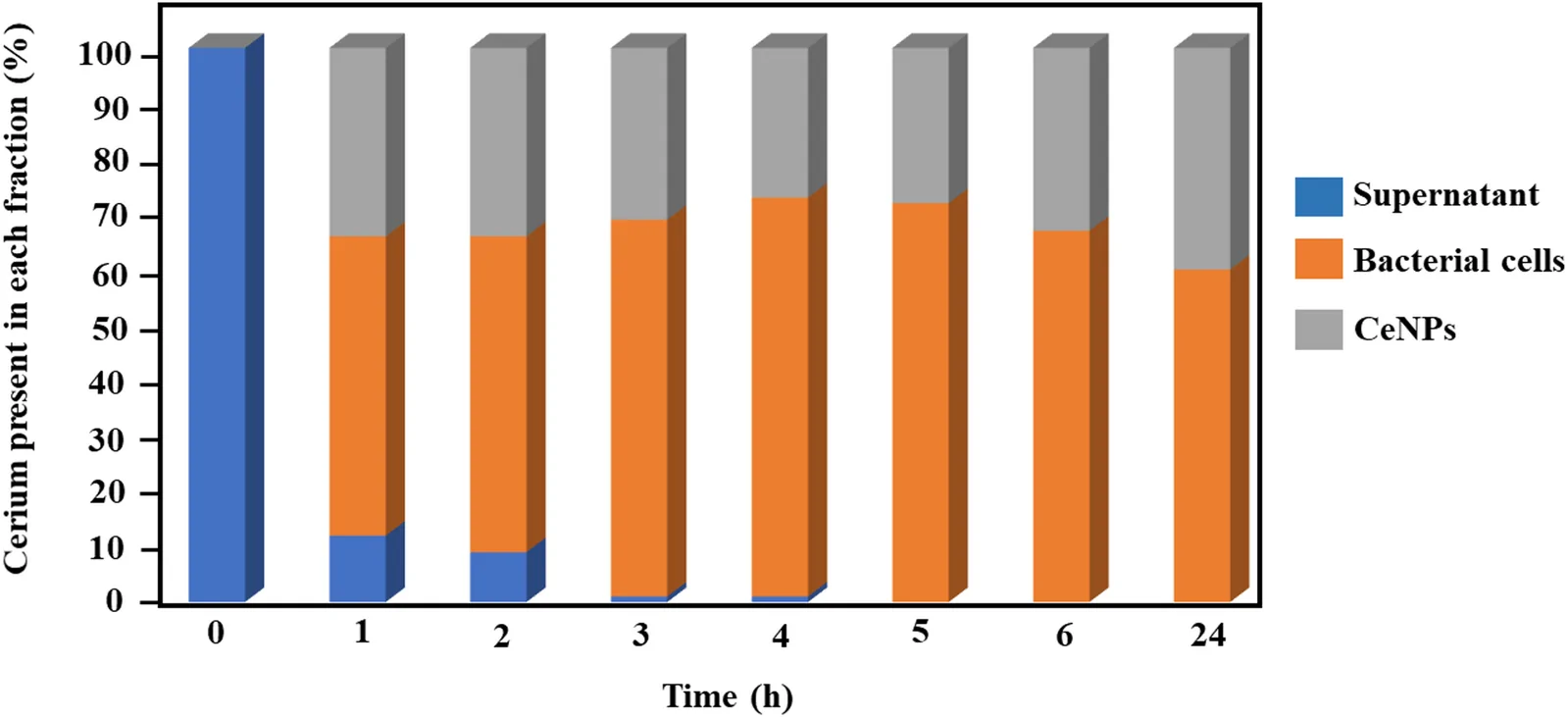
**Ce removal from solution by** ***P. putida*** **KT2440.** Distribution of Ce (in percentage) in the supernatant (blue), associated with bacterial cells (orange), and converted into CeNPs (gray) at different time points following exposure of *P. putida* KT2440 resting cells to 0.1 mM CeCl₃. The data presented represent the mean of three independent experiments.

To investigate the temporal transformation of soluble Ln in the presence of *P. putida* KT2440 cells under non-growing, resting conditions we assessed the depletion of Ce³⁺ from a saline buffer (0.85% NaCl) containing 0.1 mM CeCl₃, the highest concentration that did not significantly impair bacterial viability (Fig. 1). Bacterial suspensions were incubated at 30 °C for 24 h, and the residual Ce³⁺ in the supernatant was quantified at multiple time points using inductively coupled plasma-optical emission spectrometry (ICP-OES), as detailed in Materials and Methods. A rapid reduction in soluble Ce³⁺ was observed, with nearly 80% removed in the first hour and almost 98% within the first 3 h of incubation. Ce³⁺ became nearly undetectable after 5 h (Fig. 2). Similar results were observed when Gd instead of Ce was supplied to the medium (data not shown). This is, to our knowledge, the first report of Ln transformation kinetics under resting cell conditions. Two non-mutually exclusive mechanisms may explain this fast depletion: (i) transformation of Ce³⁺ into insoluble chemical species (e.g., cerium phosphate or cerium nanoparticles) that are no longer detectable in ionic form, or (ii) adsorption and/or nucleation of Ce³⁺ on the bacterial cell surface. To assess the bioaccumulation capacity and metal recovery potential, bacterial pellets were collected post-incubation, washed to eliminate unbound ions, and subjected to acidic extraction as described in detail in Material and Methods section. Finally, the Ln content in the acid-solubilized fraction was quantified by ICP-OES, enabling estimation of metal recovery yield and adsorption kinetics. Figure 2 shows that *P. putida* KT2440 adsorbs around 70% of the Ce present on the medium, which can be due to the phosphorous groups located at the external part of the cell envelope. This adsorption starts very fast, since more than 50% of Ce was located on cell pellets after 1 h incubation. The adsorption increases for few more hours and raise the maximum at 5 h of exposure. The number of bacterial cells (6 × 10^8^ CFU mL^− 1^) remained almost constant as observed previously (Fig. 1).

Previous studies have demonstrated Ln surface interactions under microbial growth conditions, for instance, amorphous phosphate-bound Ln precipitates have been found on the surface of *Saccharomyces cerevisiae* exposed to Ce and Yb^39^ and *Bacillus licheniformis* has been shown to bioaccumulate Ce³⁺ at the cell surface^40^. However, to our knowledge, this is the first report on Ln transformation kinetics by bacteria under resting cell conditions.

Whereas the use of extremophilic bacteria for Ln recovery is extended^41^, few examples of non-genetically modified mesophilic bacteria useful for the adsorption and recovery of Ln from solution (with an efficiency of nearly 50%) have been reported^42,43^. Here we describe that the mesophilic and environmental model bacterium *P. putida* KT2440 displays the ability to recover 70% of the Ce from solution in a few hours of exposure, hence offering significant advantages over previously described bacteria with lower adsorption efficiencies. The high sequestration rate presented by KT2440 suggests a highly efficient surface binding or intracellular uptake mechanism, which could be leveraged for Ln bioremediation or bio-recovery from electronic waste or mining effluents. These unique adaptations in cell wall composition, extracellular polymeric substances, or transport systems should be further characterized and used as a target for potential genetic or metabolic engineering in the future.

Notably, part of the recovered Ce from solution is present in non-ionic forms since the ICP technique is not able to detect its presence, suggesting the formation of CeNPs. Moreover, the amount of CeNPs remained constant (around 30% of the initial amount of Ce) along the incubation time (Fig. 2). Extracellular Ln mineralization as crystalline nanowires was found on the surface of some strains of *Pseudomonas stutzeri*^44^, and *Pseudomonas kunmingensis* was reported to facilitate in situ nanocrystal formation with several Ln (La to Gd)^45^. However, the physicochemical characterization of these nanostructures and the mechanistic insights into their formation remain largely unexplored.

### Production of CeNPs and GdNPs by *P. putida* KT2440

**Fig. 3:**
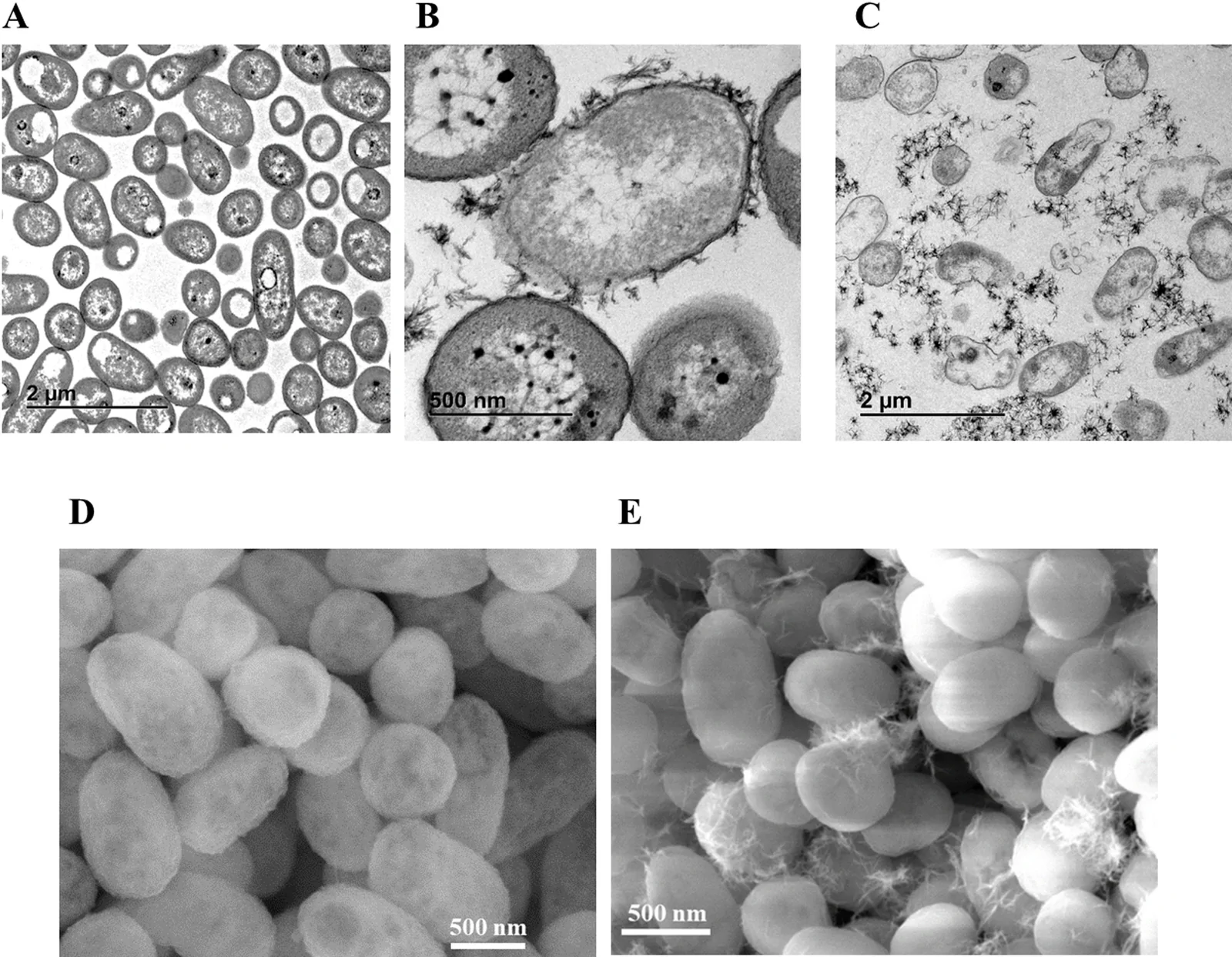
**Production of CeNPs by** ***P. putida*** **KT2440.** TEM (**A**, **B**, **C**) and SEM (**D**, **E**) analyses showing CeNPs outside of cells exposed to 0.1 mM CeCl_3_ (**B**, **C**, **E**) in comparison with cells without exposition to Ce (**A**, **D**). The incubation time were 3 h (**A**, **B**, **D** and **E**) or 24 h (**C**).

**Fig. 4:**
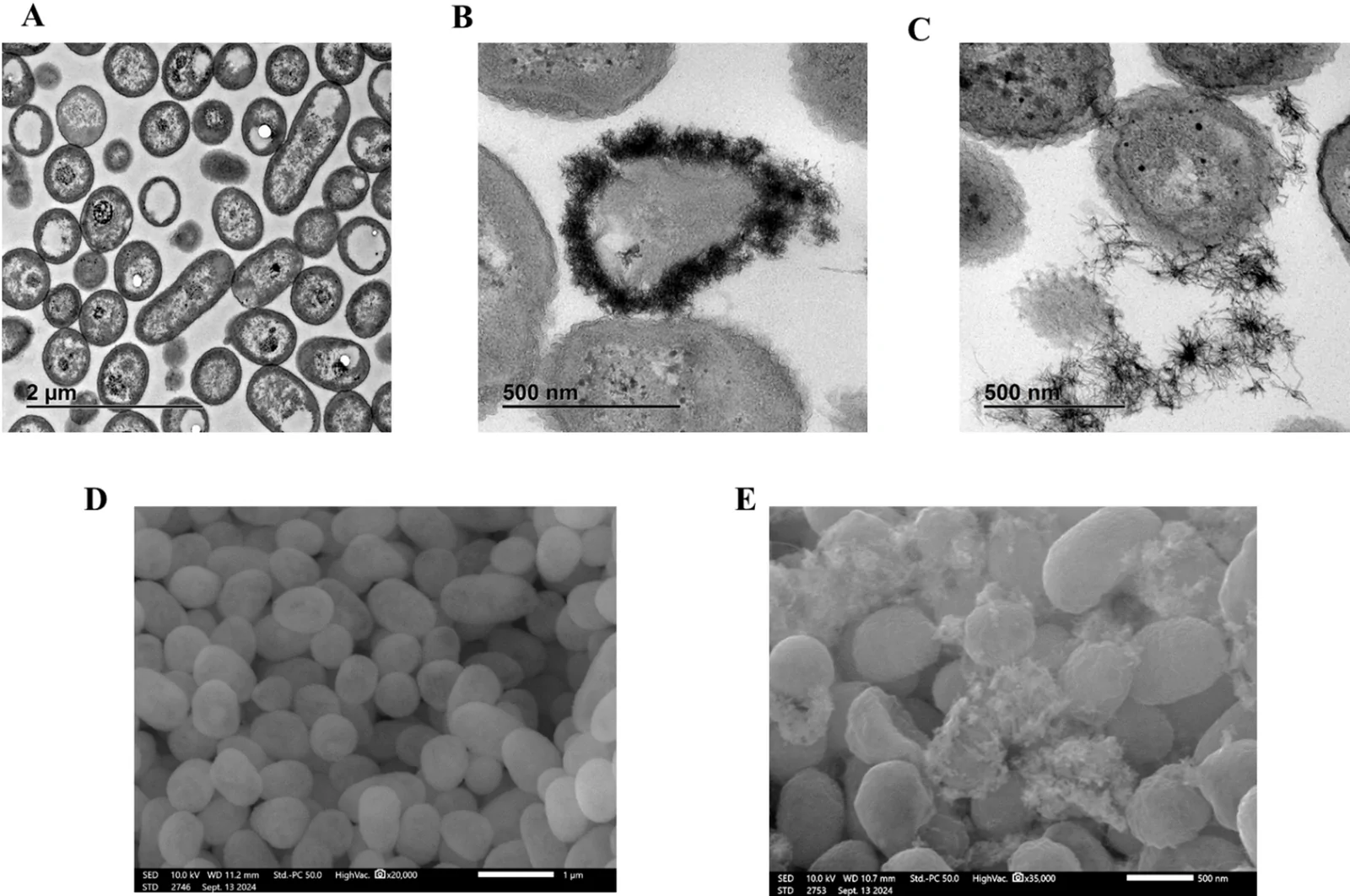
**Production of GdNPs by** ***P. putida*** **KT2440.** TEM (**A**, **B**, **C**) and SEM (**D**, **E**) analysis showing GdNPs outside of cells exposed to 0.1 mM GdCl_3_ (**B**, **C**, **E**) in comparison with cells without exposition to Gd (**A**, **D**). The incubation time were 3 h (**A**, **B**, **D** and **E**) or 24 h (**C**).

As indicated above, *P. putida* KT2440 cells may convert part of soluble Ln into LnNPs. To investigate the ability of *P. putida* KT2440 to mediate the formation of CeNPs and GdNPs, cells were sequentially cultured in LB, transferred to minimal medium, and subsequently resuspended in saline solution at an *OD*_*600*_ of 1.2 (≈ 10⁹ cells/mL) and exposed to 0.1 mM CeCl₃ or GdCl₃ during 24 h. At each time point, culture pellets were collected and processed for electron microscopy. In both Ce- and Gd-exposed cells, electron-dense deposits were observed by TEM and SEM at short incubation times (3 h), closely associated with the cell envelopes and forming dense structures outlining the cell periphery (Figs. 3B and E and 4B and E), which were absent in untreated controls (Figs. 3A and 4A). Although these results strongly suggest that initial nucleation occurs at the cell surface, a contribution of cytoplasmic or periplasmic processes cannot be ruled out. With prolonged incubation times (24 h), an increasing number of lysed cells was observed, consistent with the release of both CeNPs and GdNPs from the cells surface (Figs. 3C and 4C). Together, these observations indicate a shared mechanism for Ln nanoparticle biosynthesis in *P. putida* KT2440, in which intact cells favor surface-associated nanoparticle accumulation, while extended exposure promotes partial lysis and subsequent release into the surrounding medium.

The formation of Ln-complexes in the periplasm or at the cell surface may represent a strategy to mitigate Ln toxicity. By sequestering ionic forms of the metal into insoluble complexes, cells reduce the likelihood of these ions interfering with essential cellular functions. Over time, these complexes may increase in structural complexity, ultimately forming crystalline assemblies that display properties characteristic of nanoparticles. Based on our current data, we consider that the LnNPs formation is consistent with a biomineralization-like mechanism. Specifically, resting cells of *P. putida* remain metabolically active at a basal level, which may contribute to the release of phosphate and the establishment of a chemically reactive microenvironment at the cell surface. Notably, nanoparticle formation was not observed when cells were inactivated (autoclaved) (data not shown), suggesting that intact and/or physiologically active cells are required, and supporting a cell-dependent process beyond simple passive precipitation on dead biomass. Although the formation of LnNPs is consistent with a biomineralization-like process, alternative mechanisms potentially contributing to phosphate release should also be considered. Some lanthanide-based materials have been reported to exhibit catalytic and enzyme-mimetic activities, including phosphatase-like and redox-related behavior under certain conditions^46^. Such activities could potentially promote the hydrolysis or degradation of phosphorus-containing biomolecules, including phospholipids, phosphate esters, or nucleic acid-related compounds, thereby contributing to local phosphate availability for lanthanide phosphate nucleation^47^. However, the nanoparticles formed by *P. putida* KT2440 are consistent with lanthanide phosphate rather than CeO₂-like nanostructures, that are typically associated with strong Ce³⁺/Ce⁴⁺ redox cycling and nanozyme activity^47^. Reports on bacterial bioproduction of crystalline or amorphous lanthanide structures are very scarce. Periplasmic deposits of lanthanum were first described in the methylotrophic *Beijerinckiaceae* bacterium RH AL1^16^; however, that study did not assess the physiological impact of Ln accumulation on the bacterium. More recently, the biosynthesis of electron-dense nanostructures composed of Na, Y, F, Yb, and Er was observed in the Antarctic bacterium *Shewanella baltica*, where they were localized in the periplasm, adhered to the outer membrane, and dispersed extracellularly^17^. As mentioned before, some extracellular Ln crystalline nanowires were found on the surface of some strains of *P. stutzeri*^44^, and *P. kunmingensis* was^45^ without any physicochemical characterization of these nanostructures. Also, fungi such as *S. cerevisiae* produce nanostructures of different Ln when the cells grew in their presence^39,48^.

Considering that *P. putida* KT2440 represents one of the few known examples of LnNP bioproduction, we next analyzed the properties of the CeNPs and GdNPs generated by this strain in comparison with those synthesized by conventional chemical methods.

### CeNPs characterization

**Fig. 5:**
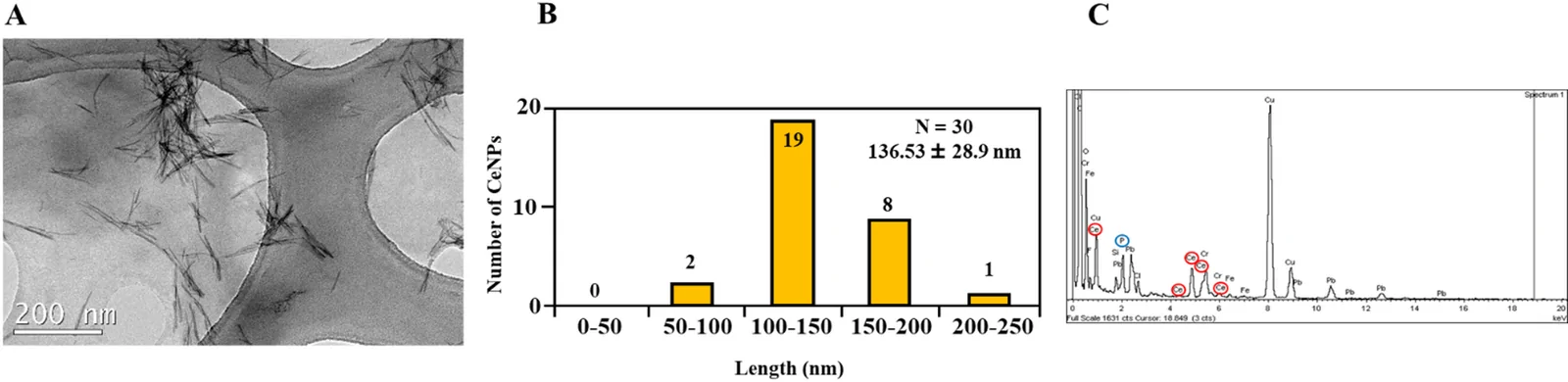
**Analysis of purified CeNPs synthesized by** ***P. putida*** **KT2440.** TEM analysis (**A**), size distribution calculated using ImageJ software (**B**) and EDX analysis showing Ce (red circles) and phosphorus (blue circle) elements (**C**) of CeNPs produced by *P. putida* KT2440 resting cells exposed to 0.1 mM CeCl_3_ during 12 h.

We analyzed the properties of the CeNPs generated by *P. putida* KT2440 resting cells after 12 h of exposure to Ce. CeNPs were purified using protocols previously optimized in our laboratory for selenium and tellurium nanostructures^49^. TEM revealed that the purified CeNPs adopted a nanorod morphology (Fig. 5A), with average dimensions of 136.53 ± 28.9 nm in length and 5.2 ± 0.9 nm in width. (Fig. 5B) Energy-dispersive X-ray spectroscopy (EDX) analysis of CeNPs revealed the presence of phosphorus, suggesting that their chemical composition was cerium phosphate (Fig. 5C). Although the experiments were conducted in 0.85% NaCl solution without added phosphate, several biological sources can account for the presence of phosphate required for CePO₄ formation. First, bacterial cells inherently contain significant amounts of phosphorus in the form of intracellular pools (e.g., polyphosphates, nucleic acids, and phosphorylated metabolites) as well as membrane-associated phospholipids. Even under resting conditions, bacteria can release phosphate into the surrounding medium through basal metabolic activity and turnover processes^50^. Furthermore, partial cell lysis over the 24 h incubation period cannot be excluded and may also contribute to phosphate release. Therefore, the phosphate required for lanthanide phosphate formation might originates from a combination of basal metabolic phosphate release and limited cell lysis. These processes can generate sufficient phosphate concentrations at the cell surface to promote biomineralization.

**Fig. 6:**
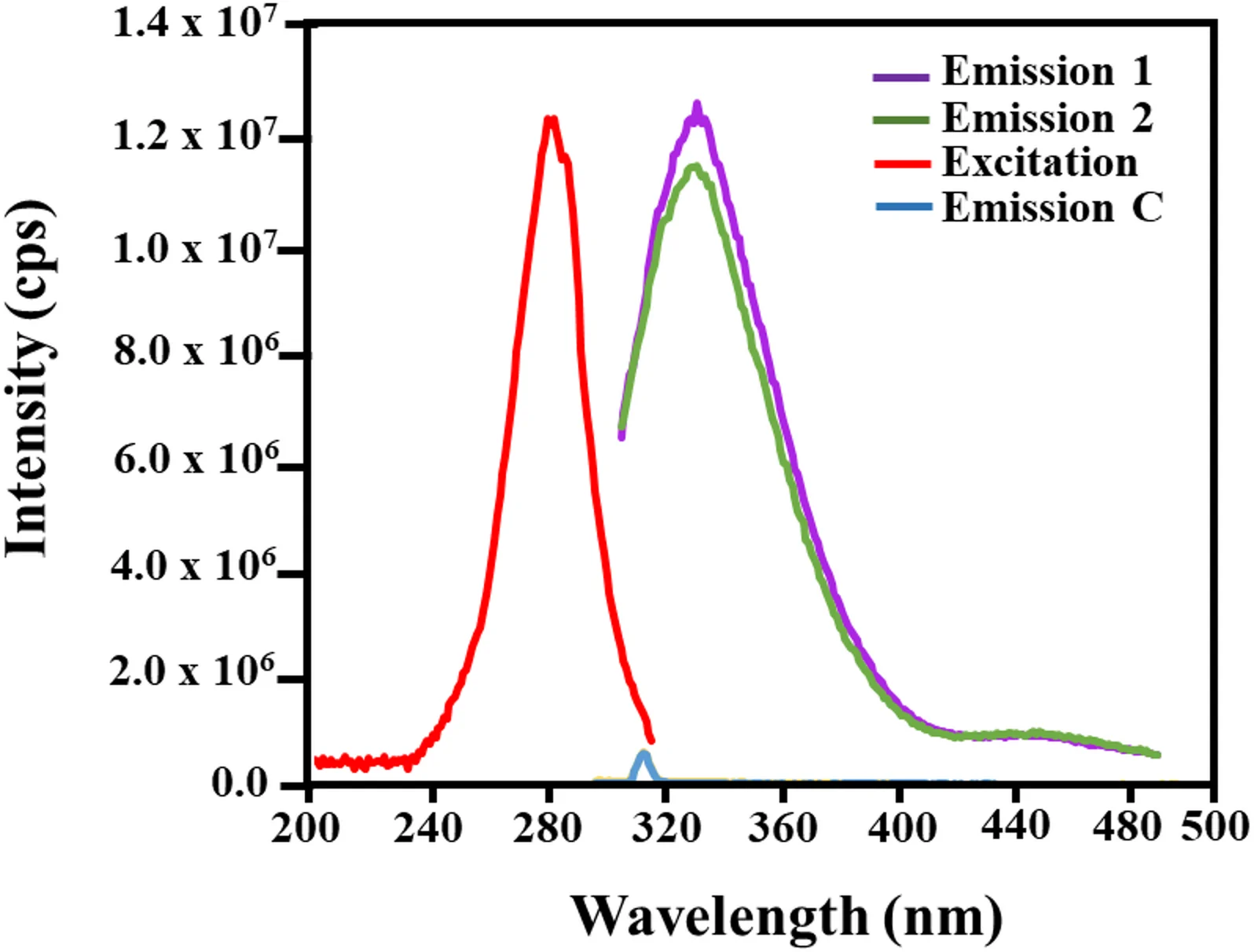
**Photoluminiscence analysis of CeNPs produced by** ***P. putida*** **KT2440.** Samples of 400 µL of water containing a suspension of CePO_4_NPs were analyzed in a modular spectrofluorometer. The record emission of duplicated samples (Emission 1 and Emission 2) and excitation profiles (Excitation) are plotted in colors indicated in the legend. Control emission profile (Emission C) was obtained with the blank (water) under the same experimental conditions. Fluorescence intensity was expressed in counts per second (cps, corresponding to photons per second).

Cerium phosphate nanoparticles (CePO₄NPs) are well recognized for their unique optical features, particularly their tunable luminescence^51,52^. CePO₄-based nanostructures are an important functional family, with demonstrated utility in catalysis^53^, high-temperature stability^54^, UV absorption for sunscreen formulations^55^, and electrochemistry^56^. Moreover, hydrothermally synthesized CePO₄ nanowires exhibit photoluminescence upon excitation at around 330 nm, emitting in the near-UV/blue region around 350 nm^51,57^. However, to our knowledge, the photophysical properties of bioproduced CePO₄NPs have not yet been investigated. To determine whether biogenic CePO₄NPs retain the photophysical behavior of their chemically synthesized counterparts, we performed photoluminescence assays using a modular spectrofluorometer. Purified nanoparticle suspensions (400 µL per assay) were analyzed, and spectra were normalized to the maximum excitation intensity. The sample was initially spectrophotometrically scanned, revealing an excitation peak at 287 nm. The excitation wavelength was then fixed at 287 nm to record emission profile within the range of 300–500 nm (a spectral window of approximately 15 nm was maintained between the excitation wavelength and the onset of emission). An emission maximum was observed at 335 nm. Subsequently, an excitation profile was acquired between 200 and 320 nm while fixing the emission wavelength at 335 nm, resulting in a maximum excitation peak at 286 nm (Fig. 6). Control emission profiles were obtained with the blank (water) under the same experimental conditions, i.e., excitation wavelength at 287 nm and emission recorded between 300 and 700 nm. Data from the blank beyond 500 nm were excluded. These values are in agreement with those reported for chemically synthesized CePO₄NPs^58,59^, indicating that the bacterial process preserves the intrinsic photoluminescent properties of CePO₄. To our knowledge, this constitutes the first demonstration that CePO₄NPs produced by bacteria display stable photoluminescence. Such findings not only confirm the structural fidelity of biogenic CePO₄NPs relative to their chemically synthesized counterparts but also highlight their potential for biotechnological exploitation. For instance, CePO₄NPs can be doped with additional lanthanide or transition-metal ions to modulate emission spectra and achieve tailored photonic responses (52, 60). Extending these strategies to biosynthesized materials could provide a sustainable route to novel luminescent nanophosphors with applications in bioimaging, sensing, photocatalysis, and optoelectronics.

## GdNPs characterization

**Fig. 7:**
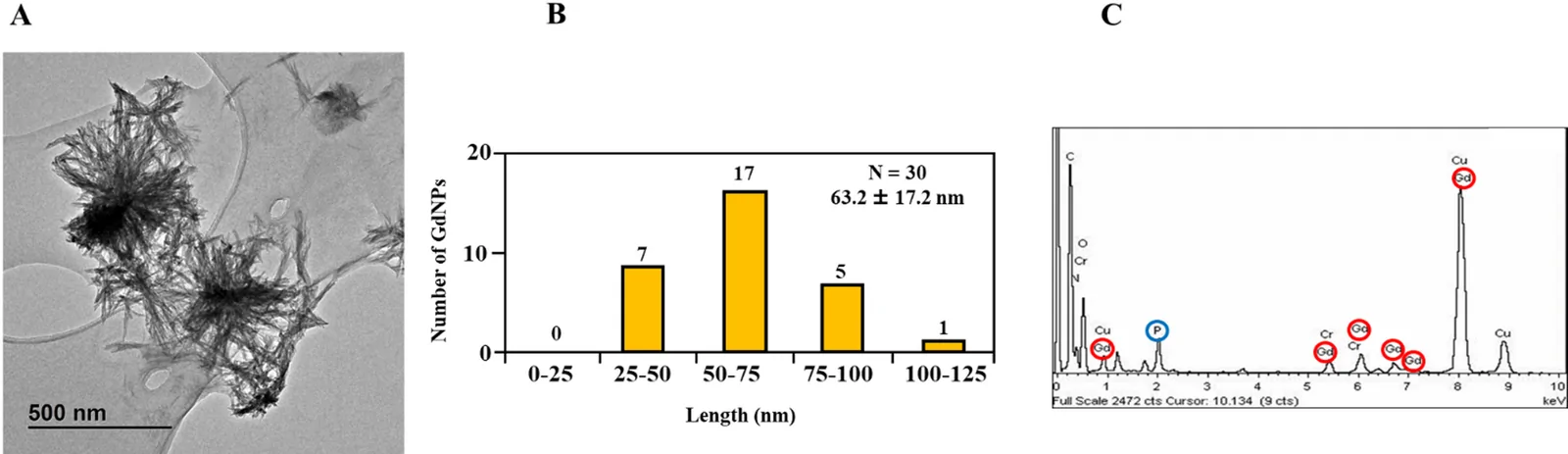
**Analysis of purified GdNPs synthesized by** ***P. putida*** **KT2440.** TEM analysis (**A**), size distribution calculated using ImageJ software (**B**) and EDX analysis showing Gd (red circles) and phosphorus (blue circle) elements (**C**) of GdNPs produced by *P. putida* KT2440 resting cells exposed to 0.1 mM GdCl_3_ during 12 h.

**Fig. 8:**
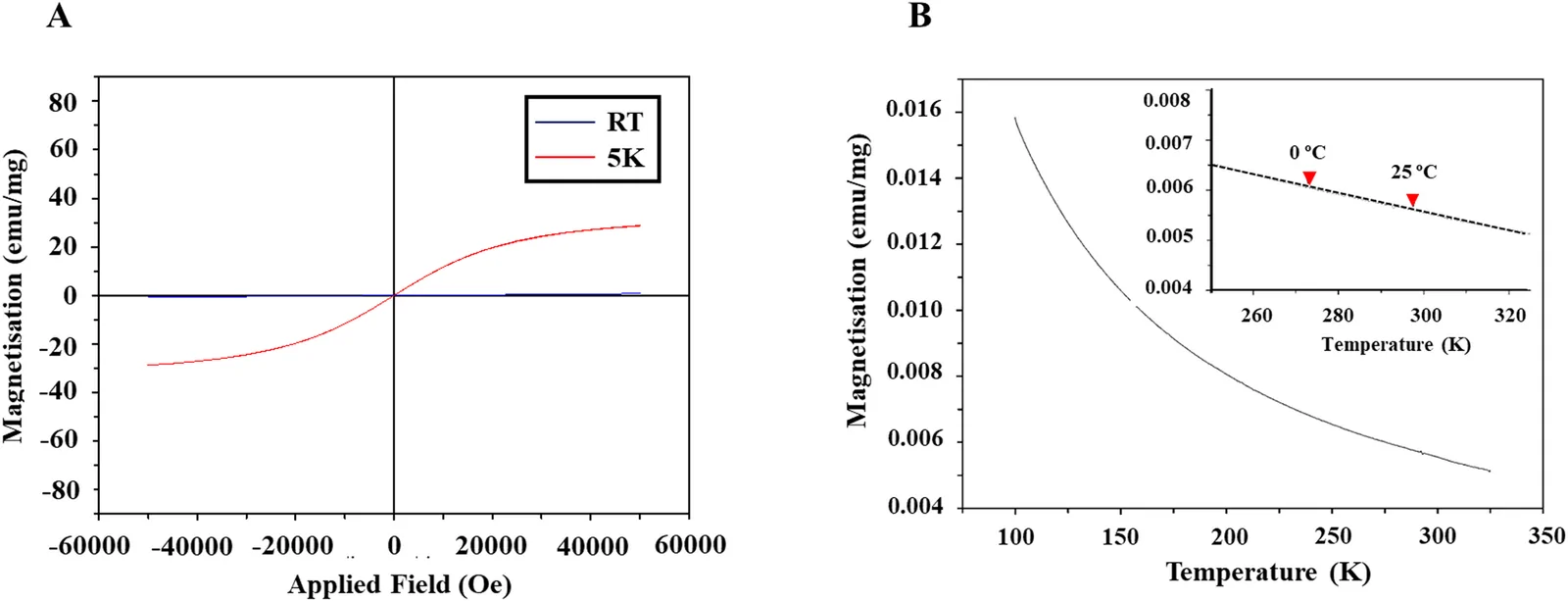
**Magnetic analysis of GdNPs produced by** ***P. putida*** **KT2440.** (**A**) Magnetization curves of GdPO_4_NPs measured at room temperature (RT) and at 5 K. (**B**) Variation of the magnetization of GdPO_4_NPs measured at temperatures between 100 and 325 K with magnification of the results at temperatures close to the room temperature.

GdNPs bioproduced by *P. putida* KT2440 were purified using protocols previously described^49^. TEM revealed that the purified GdNPs adopted a nanorod morphology (Fig. 7A), with average dimensions of 63.2 ± 17.2 nm in length and 7.1 ± 1.2 nm in width. (Fig. 7B) Energy-dispersive X-ray spectroscopy (EDX) analysis of GdNPs revealed the presence of phosphorus, suggesting that their chemical composition was gadolinium phosphate (Fig. 7C). Gadolinium phosphate nanoparticles (GdPO₄NPs) exhibit several physicochemical and functional properties that make them attractive for biomedical and photonic applications^61^. GdPO₄NPs, owing to the high magnetic moment of Gd³⁺ ions and their robust paramagnetic response, have emerged as promising candidates in advanced biomedical and technological applications, as magnetic resonance imaging (MRI) contrast agents^62^. At ultralow temperatures, GdPO₄ exhibits frustrated antiferromagnetic ordering, which has been exploited for magnetic refrigeration, highlighting its potential in cryogenic cooling technologies^63^. To determine whether biogenic GdPO₄NPs retain such paramagnetic behavior, we measure the magnetization curves at room temperature and at 5 K. At room temperature, GdNPs exhibit paramagnetic behavior, while at low temperatures, the reduced thermal agitation allows atomic magnetic moments to align more easily with the external magnetic field resulting in a strengthen of the magnetic properties (Fig. 8A). The smooth variation of the magnetization with temperature at around 20 °C reveals that there is not a transition from a paramagnetic to a ferromagnetic behavior, supporting the fact that the biogenic material is most likely a paramagnetic GdPO_4_^66^(Fig. 8B).

## Conclusions

In the present study, we provide evidence that *P. putida* KT2440 can be harnessed for the simultaneous removal of Ce and Gd from solution and the biogenic production of CePO₄NPs and GdPO_4_NPs, respectively. Beyond their biogenic origin, these nanoparticles were shown to retain intrinsic photoluminescent (CeNPs) and paramagnetic (GdNPs) properties comparable to those reported for their chemically synthesized counterparts. To our knowledge, this is the first report demonstrating that LnPO₄NPs produced in an environmentally friendly manner display measurable luminescence or paramagnetic abilities. The demonstration that biosynthesized LnPO₄NPs maintain functional and chemo-physical behavior similar to that of chemically synthesized LnPO₄NPs opens several promising avenues. First, the process validates *P. putida* KT2440 as a candidate platform for scalable, sustainable LnNPs production. Second, biogenic synthesis could facilitate novel material properties: LnNPs generated in biological environments may incorporate organic moieties or unique surface chemistries that enhance stability, dispersibility, or functionalization potential. Third, and perhaps most importantly, biosynthetic processes can be integrated with circular economy strategies. For example, Ln-containing waste streams from industry or electronics recycling could serve as feedstocks for bacterial nanoparticle biosynthesis, simultaneously contributing to waste valorization and material recovery.

Future work should focus on optimizing bioprocess parameters to improve nanoparticle yield, size uniformity, and reproducibility. Equally important will be the exploration of doping strategies, in which additional REEs or transition metals are incorporated into biogenic LnPO₄NPs to fine-tune their optical or catalytic properties. Finally, translational studies assessing the scalability of such bioprocesses and their integration into existing waste management or nanomaterial production pipelines will be crucial to fully realize their potential.

## Methods

### Bacterial strains, culture media and growth conditions

*P. putida* KT2440 strain derived from the TOL plasmid-cured *P. putida* mt-2 strain^65^. *P. putida* KT2440 strain was grown at 30 °C with vigorous shaking (200 rpm) in rich lysogeny broth (LB) or M63 minimal medium^66^ with 2 mM MgSO_4_ and 0.2% (w/v) glucose as the sole carbon source.

### Determination of Ce and Gd toxicity

The *P. putida* KT2440 cultures were grown in LB overnight at 30 °C. Next day, cultures were transferred to fresh media in M63 minimal medium supplemented with 0.2% glucose at initial *OD*_*600*_ of 0.1 in a final volume of 50 mL. Cultures in stationary phase (*OD*_*600*_ 2.0) were centrifugated for 20 min at 3800 rpm at room temperature, and then the supernatant was discarded. The cell pellet was washed three times with saline solution (NaCl 0.85%). The final pellet was then resuspended in saline solution to a final *OD*_*600*_ 1.3–1.5 supplemented with Ln chloride, cerium (Ce) or gadolinium (Gd) at final concentrations of 0, 0.05, 0.1, 0.25, 0.5 and 1 mM. The pH of the unbuffered saline suspension containing lanthanide chlorides and bacterial cells was monitored during incubation, showing an initial value of approximately 5.5 that gradually increased to around 6.0 after 24 h. Samples were taken at different times (0, 3, 4, 5, 6 and 24 h) to check the Ln toxicity by using viable counts in LB plates (serial dilutions between 10^− 1^ and 10^− 6^). Colony forming units (CFU) were counted after 24 h of incubation at 30 °C.

### Cerium mass balance determination

Total cerium (Ce_total) was calculated from the initial concentration in the culture medium. After incubation, the culture was centrifuged to separate the supernatant and the bacterial pellet. Cerium in the supernatant (Ce_supernatant) was directly quantified by ICP-OES. The bacterial pellet was subjected to acid digestion using 0.7 ml nitric acid (65% w/w for analysis grade) during 2 h and H_2_O_2_ (30% high pure grade) during 12 h. After that time, 9 ml of H_2_0 was added and the resulting cerium concentration (Ce_pellet) quantified by ICP-OES represents the total cerium associated with the biomass (including both adsorbed and intracellular cerium). The fraction attributed to “CeNPs” was estimated by difference as:

[Ce_NPs = Ce_total − (Ce_supernatant + Ce_pellet)]

This fraction therefore corresponds to non-recovered cerium, which we interpret as cerium present in particulate form not accounted for in the soluble or biomass-associated fractions, potentially due to the presence of extracellular nanoparticulate material.

### Production of Ce and Gd nanoparticles

The production of cerium nanoparticles (CeNPs) and gadolinium nanoparticles (GdNPs) was performed in resting cells. For that *P. putida* KT2440 cells resuspended in saline solution were obtained in the conditions described in the previous section and mixed with 0.1 mM of CeCl_3_ or GdCl_3_. After different times of incubation at 30 °C with the CeCl_3_ or GdCl_3_, the cultures were centrifuged to separate pellets from supernatants. Removal of cerium and gadolinium from the supernatants was determined by inductively coupled plasma optical emission spectroscopy (ICP-OES) (Perkin Elmer Optima 2100 DV). Cell pellets were resuspended in water and analyzed by TEM searching for the presence of CeNPs or GdNPs.

### Purification of CeNPs and GdNPs

For the purification of the CeNPs and GdNPs a protocol previously optimized in our laboratory for selenium and tellurium NPs^49^ was followed. This procedure is based on a separation by centrifugation of the NPs produced by the bacteria in a mixture composed by chloroform, ethyl alcohol and water (3:1:4).

### Characterization of CeNPs and GdNPs

The shape of CeNPs and GdNPs was analyzed using electron microscopy and the size was determined by using the ImageJ software. For field emission Scanning Electron Microscopy (SEM), the CeNPs and GdNPs were filtered through 0.2 μm pore-size filters and successively dehydrated with acetone/water mixtures of 30, 50, 70, 80, 90 and 100% acetone. After critical point drying, samples were coated with graphite and gold and examined with a JEOL JSM-6330 F microscope. For Transmission Electron Microscopy (TEM) analysis, bacterial pellets containing CeNPs or GdNPs were resuspended in 1 mL sterile water and the samples were prepared by placing drops onto carbon-coated copper grids and allowing solvent to evaporate. The TEM observations were performed on a JEOL model JEM-2100 instrument operated at an accelerating voltage of 200 kV. To prepare ultrathin sections of bacteria containing CeNPs or GdNPs, bacteria were fixed in a solution containing 4% paraformaldehyde and 2.5% glutaraldehyde in 0.1 M phosphate buffer pH 7.3 (Karnovsky solution) for 4 h at 4 °C and then post-fixed for 1 h in a 1% solution of osmium tetroxide. Bacteria pellets were dehydrated in a graded series of acetone dilutions, embedded in Spurr’s resin: Spurr´s resin: acetone (1:3) for 1 h, Spurr´s resin: acetone (1:1) for 1 h, Spurr´s resin: acetone (3:1) for 2 h, 100% Spurr´s resin overnight, and then polymerized at 65 °C for 48 h. Ultrathin Sect. (60 nm) were prepared using a Leica EM UC7 ultramicrotome and stained with uranyl acetate 2% followed by Reynolds lead citrate. Electron micrographs were obtained using a JEOL 1400 plus electron microscope operated at 100 kV and equipped with a CCD Gatan Orius SC 200. The energy-dispersive X-ray spectroscopy (EDX) elemental analyses were performed using a detector (Super-X) with 0.23 sr solid angle at the same microscope. All electron microscopy observations were carried out at the Centro Nacional de Microscopía Electrónica, CNME-UCM, Madrid, Spain.

### Photoluminiscence analysis of CeNPs

The photoluminiscence properties of the CeNPs were analyzed using a modular spectrofluorometer (Fluorolog, Jobin Yvon-Spex). Purified NPs suspensions in distilled water (400 µL per assay) were used, and spectra were normalized to the maximum excitation intensity. The blank used (water) was repeated after thorough cleaning of the cuvette to rule out artifacts caused by contamination; however, the resulting profile was identical to the initial measurement. Fluorescence intensity was expressed in counts per second (cps), corresponding to photons per second. The lamp voltage applied during the measurements was 950 V.

### Magnetic characterization of the GdNPs

The magnetic behavior of the GdNPs was measured in a SQUID magnetometer (Quantum Design MPMS-5) applying a maximum field of 5 Tesla. Samples were measured in powder, packed in a gelatin capsule to measure the hysteresis loop at room temperature and at 5 K and the variation of the magnetization as a function of temperature in the temperature range between 325 and 100 K.

## Data Availability

All data generated or analysed during this study are included in this published article.
